# Efficacy and safety of Dentoxol® in the prevention of radiation-induced oral mucositis in head and neck cancer patients (ESDOM): a randomized, multicenter, double-blind, placebo-controlled, phase II trial

**DOI:** 10.1007/s00520-020-05358-4

**Published:** 2020-04-08

**Authors:** Rajesh V. Lalla, Sebastián Solé, Sergio Becerra, Claudia Carvajal, Piero Bettoli, Hernán Letelier, Alejandro Santini, Lorena Vargas, Alexander Cifuentes, Francisco Larsen, Natalia Jara, Jorge Oyarzún, Richard Feinn, Eva Bustamante, Benjamín Martínez, David Rosenberg, Tomas Galván

**Affiliations:** 1 Universityof Connecticut Health, Farmington, CT USA; 2 Clinica IRAM, Santiago, Chile; 3 Instituto Nacional del Cáncer, Santiago, Chile; 4 Fundación Arturo López Pérez, Santiago, Chile; 5 Hospital Base Valdivia, Valdivia, Chile; 6 Centro Oncologico de Antofagasta, Antofagasta, Chile; 7 Quinnipiac University, Hamden, CT USA; 8 Universidad Mayor, Santiago, Chile; 9 Ingalfarma, SpA, Santiago, Chile

**Keywords:** Oral mucositis, Radiation therapy, Head and neck cancer, Dentoxol, Mouthrinse

## Abstract

**Purpose:**

The aim of this study was to assess the efficacy and safety of Dentoxol mouthrinse in reducing the severity of oral mucositis (OM) secondary to radiation therapy (RT) for head and neck cancer.

**Methods:**

A randomized, double-blind, placebo-controlled, multicenter phase II clinical trial was conducted. Subjects were asked to use Dentoxol (*n* = 55) or control (*n* = 53) mouthrinse 5 times/day during RT. Twice a week, OM was assessed clinically using the WHO scale and the Oral Mucositis Daily Questionnaire (OMDQ) was completed.

**Results:**

The incidence of severe OM was 40.7% in the Dentoxol group and 51% in the control group (*p* = 0.265). Comparing all recorded clinical assessments, severe OM was seen in 13.3% of all assessments in the Dentoxol group vs. 21.8% in the control group (*p* = 0.000). There was a statistically significant lower proportion of assessments showing severe OM in the Dentoxol group at weeks 4, 5, and 6 of RT. The mean duration of severe OM was 11.95 days in the Dentoxol group vs. 14.59 days in the control group (*p* = 0.502). There was no difference between groups in mouth pain and its impact on function. The use of Dentoxol was safe and was not linked to any serious adverse events.

**Conclusion:**

The use of Dentoxol 5 times/day is safe and resulted in significantly fewer time-points with severe OM and a delay in the onset of severe OM, compared with a control rinse. A phase III clinical trial is warranted to confirm efficacy and address the limitations of this study.

## Introduction

Oral mucositis refers to erythematous and ulcerative lesions of the oral mucosa seen in cancer patients undergoing systemic chemotherapy and/or head and neck radiotherapy.

These lesions are painful and compromise nutritional intake and oral hygiene of the patient, which increases the risk for local and systemic infection [1–3]. Patients undergoing radiotherapy for head and neck cancer usually receive approximately 200 cGy daily dose of radiation, 5 days a week, for 5–7 continuous weeks. Almost all of these patients will develop some degree of oral mucositis. Two large studies in patients treated with radiotherapy for head and neck cancer showed that some degree of oral mucositis occurred in 94–96% of patients in the control group. Severe oral mucositis developed in 66% of control group patients in these two studies [4, 5].

Most patients who undergo radiation therapy for head and neck cancer cannot continue to be fed orally due to the pain caused by mucositis and often receive enteral nutrition through a gastric feeding tube or intravenously. Patients with oral mucositis have been shown to be significantly more likely to have severe pain and weight loss ≥ 5% [6]. One study showed that approximately 16% of patients who received radiation therapy for head and neck cancer were hospitalized due to mucositis [7]. Additionally, 11% of patients undergoing radiotherapy for head and neck cancer had unplanned interruptions in radiation therapy due to severe mucositis [7]. Radiation-induced oral mucositis also has a significant economic impact due to costs associated with pain management, liquid dietary supplements, placement of gastroesophageal tubes or total parenteral nutrition, management of secondary infections, and hospitalizations. Thus, mucositis is a clinically important and sometimes dose-limiting complication of cancer therapy, which can compromise continuity of the treatment.

Dentoxol® is a novel patented oromucosal liquid that is currently marketed in Chile for the management of oral mucositis. It contains eugenol, camphor, parachlorophenol, hydrogen peroxide, purified water, xylitol, sodium bicarbonate, sucralose, and peppermint essence, of which the first four were believed to be the main active components. Its main mode of action is understood to be through mechanical cleansing, moisturizing, lubrication, and mechanical stimulation of local epithelial regeneration, although some of its components may also have antimicrobial and soothing effects. Preliminary uncontrolled studies of Dentoxol® indicated that it can be beneficial in oral mucositis secondary to radiation therapy for head and neck cancer. No other prior studies of this combination have been reported. Therefore, a well-designed randomized controlled trial was indicated.

## Methods

### Study design

This was a parallel-group, double-blind, randomized, placebo-controlled clinical trial, conducted at 5 clinical sites in Chile (listed in Table 1). The main objective of the study was to determine the efficacy of Dentoxol® in reducing the severity of oral mucositis secondary to radiation therapy for head and neck cancer. The primary endpoint was the incidence of severe oral mucositis, defined as grade 3 or 4 on the World Health Organization (WHO) scale. The secondary objectives were as follows: (1) to determine the effect of Dentoxol® mouthwash on duration of severe oral mucositis, (2) to determine the effect of Dentoxol® mouthwash on pain due to oral mucositis, and (3) to assess the safety of Dentoxol® mouthwash. For these objectives, the measured variables were as follows: (1) duration of severe oral mucositis, defined as grade 3 or 4 on the World Health Organization (WHO) scale; (2) mouth pain scores, measured by an oral mucositis daily questionnaire (OMDQ); (3) recording of adverse events.

**Table 1:** Distribution of enrolled patients according to recruitment site and treatment group

Site	Dentoxol (*N* = 55)	Control (*N* = 53)	Total (*N* = 108)
Fundacion Arturo Lopez Perez	15	15	30
Clinica IRAM	14	16	30
INC (Sede Sur + Sede Norte)	19	16	35
Centro Oncologico de Antofagasta	1	1	2
Hospital Base Valdivia	6	5	11
Total	55	53	108

*INC*, Instituto Nacional del Cancer

### Recruitment, informed consent, and inclusion/exclusion criteria

Local Ethics Committee approval was obtained at each site and all subjects provided written informed consent. All procedures performed in studies involving human subjects were in accordance with the ethical standards of the local institutional research committee and with the 1964 Helsinki Declaration and its later amendments or comparable ethical standards. Patients were enrolled before the start of radiation therapy. It is standard clinical practice at the study sites for patients to receive dental evaluation and management prior to head and neck radiation therapy. Any active oral infection before or during the radiation therapy period was managed by clinical providers per the normal clinical processes. Inclusion criteria were as follows: patients scheduled to receive radiotherapy of at least 5000 cGy in at least 2 of 12 pre-specified areas in the oral cavity for cancer of the oral cavity, oropharynx, nasopharynx, hypopharynx, or larynx, with or without concomitant chemotherapy. Exclusion criteria were as follows: patients who did not sign the informed consent form, patients who previously indicated known allergy/intolerance to any component of the study or control product, patients who were or planned to be under treatment with contraindicated medications during the study period, patients under 18 years of age, and pregnant or nursing women. Contraindicated topical agents included any agent marketed for oral mucositis, steroids, antibiotics, laser therapy, and investigational agents. Contraindicated systemic agents included non-steroidal anti-inflammatory drugs (NSAIDS), steroids, amifostine, palifermin, sialogogues, granulocyte macrophage colony stimulating factor (GM-CSF), and investigational agents.

### Randomization and stratification

Eligible subjects from all study sites were randomly assigned by the central pharmacy to the Dentoxol® or placebo group in a 1:1 allocation ratio using web randomization software. Two important initial characteristics that can influence the severity of oral mucositis are (1) the location of the tumor (oral cavity/oropharynx, nasopharynx, hypopharynx/larynx) that determines the radiation fields and (2) the type of radiation therapy: intensity modulated radiation therapy (IMRT) or 3D radiation therapy (RT). To ensure that these two important initial characteristics were equitably distributed between the Dentoxol® and control groups, randomization was stratified according to these two variables, resulting in 6 strata. To ensure that the 1:1 allocation ratio to the Dentoxol® and control groups was maintained as the study progressed, a block size of 2 was used within each stratum.

### Blinding

Study investigators and other clinical staff as well as study subjects were blinded to the allocation of subjects to Dentoxol® or control. The control rinse contained purified water, xylitol, sodium bicarbonate, sucralose, and peppermint essence. It was similar to Dentoxol® in terms of color, flavor, and consistency and was packed in identical bottles, with the same labels. Neither the subjects nor the study staff at each site were aware of whether a subject received Dentoxol® or control.

### Interventions and assessments

Subjects were instructed to use Dentoxol® or control mouthwash 5 times daily, beginning on the first day of radiation therapy and ending on the last day of radiation therapy. All subjects were instructed to follow a standardized oral hygiene protocol during the study period—this consisted of brushing twice a day with fluoride toothpaste, flossing once a day, use of a baking soda/salt mouthwash, and denture care. Twice a week during the study period, a blinded and calibrated examiner conducted a clinical oral examination to assess the severity of oral mucositis using the WHO scale. The Oral Mucositis Daily Questionnaire [8] was completed at each study visit. Subjects were also asked to complete a daily form during the treatment period to document the use of the study rinse, concomitant medications, and side-effects. Fourteen subjects were treated clinically for oral candidiasis during the study period, which were equally balanced between the active and placebo groups (7 subjects each). No subjects were treated for oral viral infection during the study period.

All study staff were trained for their function on the study including training in clinical evaluations of oral mucositis and in completion of all study forms. This calibration was performed prior to enrollment of patients in the study. In addition, calibration materials were developed and provided to all sites for reference during the study period.

### Safety monitoring

The study medication use form included a question about any adverse events experienced by the subject. In addition, study staff reported any adverse events of which they were aware. All serious adverse events were reported to the local Ethics Committee according to institutional requirements. A Data and Safety Monitoring Board (DSMB) reviewed adverse events and study data at 3 milestones: after 50 subjects completed the study, after 100 subjects completed the study, and at the end of the study.

### Data quality control

Copies of completed study forms were regularly sent from each study site to a data coordination center where they were carefully reviewed to identify any missing data, inconsistencies, or other discrepancies. Any queries were sent to the study site requesting clarification of such discrepancies in a timely manner. These procedures ensured the quality of data collected and minimized missing or inconsistent data.

### Sample size

Sample size was calculated based on the primary endpoint of incidence of severe oral mucositis, defined as grade 3 or 4 on the WHO scale. Data from the control groups of two large studies of patients who underwent radiotherapy for head and neck cancer were used in planning the sample size [4, 5]. Each of these studies had 94 subjects in the control group. In both studies, 66% of control patients developed severe oral mucositis. Therefore, an expected incidence of 66% for the control group was used in the calculation of the sample size. Since no data were available on the expected incidence reduction in patients with Dentoxol®, a clinically relevant effect size was used. An absolute reduction of 25% (from 66 to 41%) in the incidence of severe oral mucositis would be clinically and economically significant. Therefore, sample size calculations were done to estimate the number of subjects needed to find a 25% reduction in the incidence of severe oral mucositis, with 80% power, when a two-tailed test is applied to a 5% significance level. Based on these parameters, it was calculated that 62 subjects per group would be needed. To account for possible withdrawals, the aim was 70 subjects per group (total of 140 subjects).

### Statistical analyses

Continuous variables were described as measures of central tendency and dispersion. Dichotomous variables were tabulated and described by absolute and relative frequencies (percentages), according to treatment group. The incidence of severe mucositis of both groups was compared by means of a test of proportions (exact of Fisher). In addition, a logistic regression model was used to evaluate the impact of Dentoxol® use on the incidence of severe oral mucositis. For the primary outcome (development of severe oral mucositis), the Cochran-Mantel-Haenszel test was used, stratified by tumor location and type of radiation therapy so as to compare the Dentoxol® and control groups. The Kaplan-Meier estimator was used to model the duration of oral mucositis between groups. A multilevel model with nested days within patients was used to model daily measurements of mouth pain score and functional limitations between groups.

## Results

**Fig. 1:**
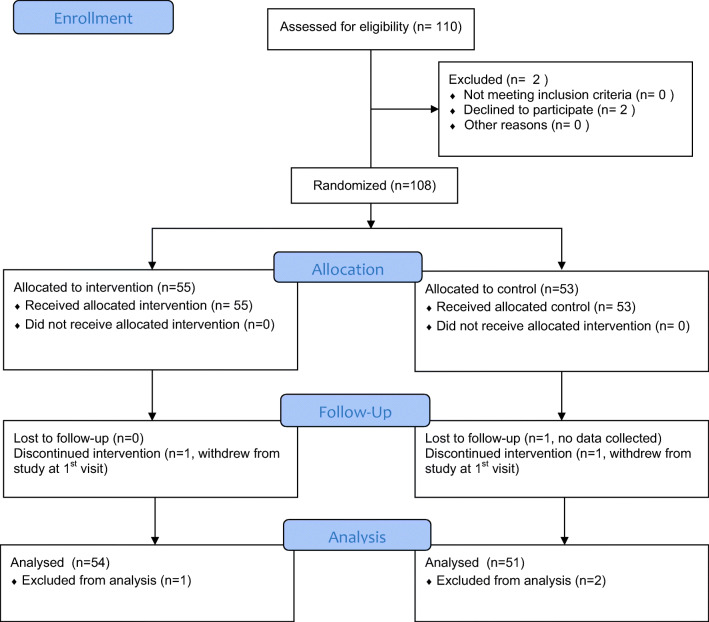
CONSORT flow diagram

Based on the results of the interim analysis, enrollment was stopped after 108 subjects had been enrolled to this study. Table 1 shows the enrollment at each site in the Dentoxol and control groups. Table 2 shows a comparison of baseline characteristics between the Dentoxol and control groups. Figure 1 shows the CONSORT flow diagram for the study.

**Table 2:** Baseline characteristics of the study sample

Variable	Dentoxol (*N* = 55)		Control (*N* = 53)		Total (*N* = 108)	
Sex						
Male	42		31		73	
Female	13		22		35	
Age*	61.21	(13.48)	61.88	(12.19)	61.54	(12.80)
Strata						
OC/Oropharynx IMRT	19		18		37	
OC/Oropharynx RT	25		24		49	
Nasopharynx IMRT	1		2		3	
Nasopharynx RT	2		1		3	
Hypopharynx IMRT	3		2		5	
Hypopharynx RT	5		6		11	
Concurrent chemotherapy						
Yes	17		10			
No	38		43			
Radiation therapy						
Median total dose (range)	66 Gy	(4–70 Gy)	66 Gy	(50–70 Gy)		
Mean total dose	64.48 Gy		65.73 Gy			
Median number of fractions (range)	33	(2–37)	33	(20–35)		

*The values for this variable correspond to the mean in years and standard deviation

*OC*, oral cavity

### Compliance with study rinse

Compliance with study rinse use was good overall with a little more than 10% of the patients rinsing less than 2 times/day, about 20% rinsing 2 to 4 times/day, and the remaining 70% of the patients rinsing more than 4 times/day. Compliance did not differ significantly between the Dentoxol and control groups (*p* = 0.360). In the Dentoxol group, the mean number of weekly doses used in weeks 1–6 ranged from 26 to 31 doses. In the control group, the mean number of weekly doses used in weeks 1–6 ranged from 30 to 32 doses.

### Incidence of severe oral mucositis

In the Dentoxol group, 22 of 54 subjects (40.7%) developed severe oral mucositis, compared with 26 of 51 subjects (51.0%) in the control group (*p* = 0.265, adjusted for strata).

When examined by week, a difference in the proportion of subjects with severe oral mucositis emerged by week 3 and continued until week 6, in favor of the Dentoxol group. This difference was statistically significant at weeks 3 (*p* = 0.007) and 4 (*p* = 0.013) and close to statistical significance at week 5 (*p* = 0.055). (Table 3).

**Table 3:** Proportion of subjects with severe oral mucositis by week

Week	Dentoxol (*n* = 55 subjects)				Control (*n* = 53 subjects)				*p* value
	Without severe oral mucositis		With severe oral mucositis		Without severe oral mucositis		With severe oral mucositis		
	*N*	%	*N*	%	*N*	%	*N*	%	
1	49	94.2	3	5.8	49	98.0	1	2.0	0.327
2	49	100	0	0.0	42	95.5	2	4.5	0.131
3	47	95.9	2	4.1	30	76.9	9	23.1	0.007*
4	42	91.3	4	8.7	29	70.7	12	29.3	0.013*
5	33	73.3	12	26.7	19	52.8	17	47.2	0.055
6	29	64.4	16	35.6	15	46.9	17	53.1	0.125
7	22	57.9	16	42.1	11	44.0	14	56.0	0.280
8	11	57.7	6	35.3	4	44.4	5	55.6	0.320

*Difference statistically significant (*p* < 0.05)

In the Dentoxol group, a total of 675 clinical assessments of oral mucositis were recorded, of which 90 assessments (13.33%) showed severe oral mucositis. In the control group, a total of 573 clinical assessments of oral mucositis were recorded, of which 125 assessments (21.82%) showed severe oral mucositis (Table 4). This difference was statistically significant (*p* = 0.0000). When all clinical assessments for oral mucositis were examined by week, it was found that there were significantly lower number of assessments showing severe oral mucositis in the Dentoxol group compared with the control group during the fourth, fifth, and sixth weeks of treatment (Table 4).

**Table 4:** Clinical assessments showing severe oral mucositis by week

Week	Dentoxol (*N* = 675 assessments)				Control (*N* = 573 assessments)				*p* value
	Without severe oral mucositis		With severe oral mucositis		Without severe oral mucositis		With severe oral mucositis		
	*N*	%	*N*	%	*N*	%	*N*	%	
1	88	100	0	0	86	100	0	0	.
2	95	100	0	0	86	100	0	0	.
3	94	96.90	3	3.09	77	91.67	7	8.33	0.191
4	84	91.30	8	8.70	58	76.32	18	23.68	0.010*
5	72	83.72	14	16.28	45	61.64	28	38.36	0.002*
6	62	73.81	22	26.19	39	55.7	31	44.29	0.026*
7	43	60.56	28	39.44	30	51.72	28	48.28	0.373
8	20	57.14	15	42.86	10	47.62	11	50	0.584
9	4	100	0	0	0	0	2	100	0.067
Total	585	86.67	90	13.33	448	78.18	125	21.82	0.0000*

*Difference statistically significant (*p* < 0.05)

Among the 17 Dentoxol subjects receiving concurrent chemotherapy, 6 subjects (35.3%) experienced severe oral mucositis. Among the 10 control subjects receiving concurrent chemotherapy, 7 subjects (70%) experienced severe oral mucositis (*p* = 0.187). On the other hand, when analyzing subjects who did not receive concurrent chemotherapy, severe oral mucositis occurred in 43.2% of such subjects in the Dentoxol group, compared with 47.5% in the control group.

**Fig. 2:**
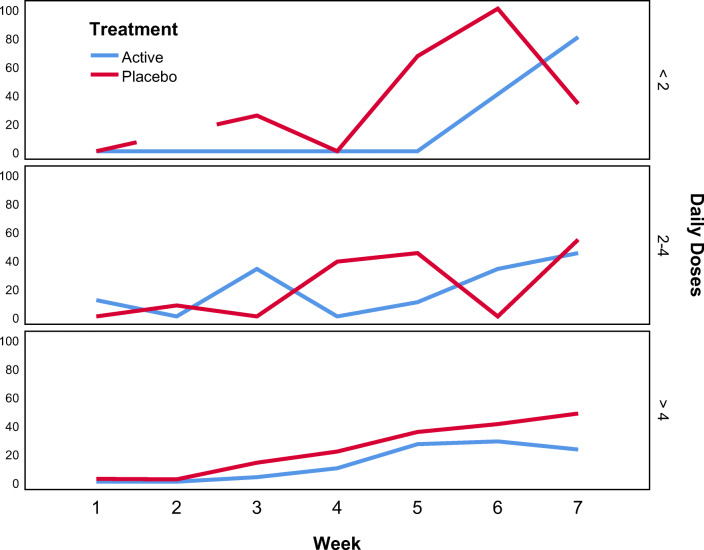
Percentage of patients with severe oral mucositis according to the number of doses and treatment group. (Note: There were no patients in the control group who rinsed less than twice/day during week 2)

Figure 2 shows the impact of compliance with study rinse use on the percentage of subjects with severe oral mucositis. The first two boxes show that in subjects who used the study rinse less often than prescribed (less than twice/day or 2–4 times/day), there was no consistent difference in the percentage of subjects with severe oral mucositis. However, among subjects who used the study rinse as prescribed (more than 4 times/day), the curves for the percentage of subjects with severe oral mucositis start to diverge after week 5, with a lower percentage of severe oral mucositis in the Dentoxol group.

### Duration of severe oral mucositis

The mean duration of severe oral mucositis was 11.95 days (standard error 3.37) for the Dentoxol group compared with 14.59 days (standard error 4.16) for the control group (*p* = 0.502). Concurrent chemotherapy had no impact on the duration of severe oral mucositis in either group.

### Pain due to oral mucositis

The Oral Mucositis Daily Questionnaire, completed daily, included questions about mouth and throat soreness and its impact on function. Overall mouth and throat soreness during the past 24 h was rated similarly in the Dentoxol and control groups (median score of 6 on a 1–10 scale in both groups). There was also no significant difference between groups for the impact of mouth and throat soreness on swallowing, drinking, eating, talking, and sleeping. Opioid pain medications were used during the study period by 49.1% of patients in the Dentoxol group and 50.9% of patients in the placebo group (*p* = 0.847).

### Adverse events

There was one serious adverse event in the Dentoxol group, a case of febrile neutropenia and sepsis. This was judged to be unrelated to study participation. There were five serious adverse events in the control group: two cases of hospitalization due to severe oral mucositis, one case of hospitalization due to pain, one case of hospitalization due to pancreatitis, and one case of hospitalization due to influenza. These were also judged to be unrelated to study participation.

The most common non-serious adverse events reported were nausea, which was reported in 25% of subjects in the Dentoxol group and 25% of subjects in the control group, and vomiting, which was reported in 13% of subjects in the Dentoxol group and 11% of subjects in the control group.

## Discussion

This phase II clinical trial was designed to assess the efficacy and safety of Dentoxol mouthwash in reducing the severity of radiation-induced oral mucositis. The primary endpoint chosen was the incidence of severe oral mucositis, defined as grade 3 or 4 on the WHO scale for oral mucositis. Dentoxol was compared with a control rinse that contained purified water, xylitol, sodium bicarbonate, sucralose, and peppermint essence. This control rinse was made of the remaining ingredients of Dentoxol after removing the ingredients that were considered active. These are hydrogen peroxide, eugenol, camphor, and parachlorophenol, which together are thought to result in mechanical cleansing and stimulation of local epithelial regeneration, along with moisturizing, lubricating, and soothing effects. This combination has not been previously studied for oral mucositis. Guidelines from the Multinational Association of Supportive Care in Cancer and the International Society of Oral Oncology (MASCC/ISOO) indicate that there is inadequate evidence for the use of commonly used saline and sodium bicarbonate rinses for oral mucositis. However, an accompanying expert opinion states that these are inert bland rinses that increase oral clearance which may be helpful for maintaining oral hygiene and improving patient comfort [9]. The study population comprised patients receiving high-dose RT for head and neck cancer. The randomization and stratification scheme used was successful in creating groups that were well balanced with respect to age, gender, tumor site, and type of RT. However, the two groups were not well balanced with respect to concurrent chemotherapy. It was originally planned to only include patients receiving concurrent chemotherapy to maintain conformity of the study population. However, in Chile, a significant proportion of head and neck RT patients do not receive concurrent chemotherapy. Therefore, this group was added for recruitment feasibility. In the Dentoxol group, 31% of the sample received concurrent chemotherapy, compared with 19% of the sample in the control group. Since concurrent chemotherapy has been demonstrated to increase the severity of radiation-induced oral mucositis in this population [6], this difference needs to be taken into account when interpreting the results of this study.

This study was significantly underpowered to achieve its initial objective of detecting a 25% absolute difference in incidence of severe oral mucositis between the two groups. Although originally planned to recruit 140 subjects (70 in each group), enrollment to this study was stopped based on the sponsor’s decision after the results of the planned interim analysis became available. At this point, 108 subjects had been enrolled to the study (55 in the Dentoxol group and 53 in the control group). Furthermore, the originally planned sample size was calculated on the basis of an expected 66% incidence of severe oral mucositis in the control group, based on the control groups of two large studies of palifermin in head and neck RT patients [4, 5]. However, we found a 47% incidence of severe oral mucositis in the control group. This may be explained by at least two factors. Firstly, the expected 66% incidence was based on a control group where 100% of subjects received concurrent chemotherapy. In our study, only a small portion of control group subjects received concurrent chemotherapy. Secondly, the placebo used in the palifermin studies was administered intravenously and can therefore be safely assumed to have no effect on severity of oral mucositis. In contrast, the placebo used in this study was an oral rinse used 5 times/day, which contained purified water, xylitol, sodium bicarbonate, sucralose, and peppermint essence. Since a major part of the action of Dentoxol is believed to be through its cleansing, moisturizing, and lubricating effects, the placebo used in this study may have resulted in some beneficial effects on severity of oral mucositis.

The use of Dentoxol resulted in a lower incidence of severe oral mucositis, compared with the control group, although this difference was not statistically significant. However, when examined by week, there was a statistically significant lower proportion of subjects with severe oral mucositis in the Dentoxol group at weeks 3 and 4 of RT. Furthermore, when all recorded clinical assessments were compared between the two groups, it was found that a significantly lower percentage of all assessments in the Dentoxol group showed severe oral mucositis compared with the control group. (*p* = 0.000). When examined by week, there was a statistically significant lower proportion of assessments showing severe oral mucositis in the Dentoxol group at weeks 4, 5, and 6 of RT. Taken together, these results demonstrate that the use of Dentoxol resulted in a significantly fewer time-points with severe oral mucositis and a delay in the onset of severe oral mucositis. The mean duration of severe oral mucositis was also 2.64 days shorter in the Dentoxol group compared with the control group, although this difference did not reach statistical significance.

Compliance with study rinse use was good with over 70% of subjects in both groups rising more than 4 times/day. Interestingly, among these subjects who used the study rinse as prescribed, the curves for the percentage of patients with severe oral mucositis start to diverge after week 5, with a declining proportion of patients with severe oral mucositis in the Dentoxol group. This suggests that compliance with the study rinse use can have an important impact on the efficacy of Dentoxol. Furthermore, as mentioned previously, there was a higher proportion of patients receiving concurrent chemotherapy in the Dentoxol group compared with the control group. This difference is likely to have negatively affected the observed overall impact of Dentoxol in this study. It is worth noting that the incidence of severe oral mucositis among the Dentoxol subjects receiving concurrent chemotherapy was half of that among the control subjects receiving concurrent chemotherapy. However, this difference did not achieve statistical significance, likely due to the relatively low numbers of subjects receiving concurrent chemotherapy.

The use of Dentoxol did not result in any significant impacts on mouth pain or on functional limitations. In keeping with ethical standards, subjects in both groups received topical anesthetics and/or systemic analgesics as clinically indicated for the management of pain due to oral mucositis. This may have confounded the measurement of any potential reductions in mouth pain and of functional limitations due to mouth pain.

The use of Dentoxol in this study was safe and was not linked to any serious adverse events. The most common non-serious adverse events seen in both groups were nausea and vomiting, which are commonly seen in this population as side-effects of cancer treatment. The incidence of these side-effects did not differ between the Dentoxol and control groups.

Strengths of this study include the prospective, randomized, double-blind, and controlled study design which minimizes the risk of bias. The randomization and stratification scheme used was successful in creating study groups that were well balanced for important determinants of mucositis severity including tumor site and type of RT. Limitations of this study include the fact that it was stopped before the planned accrual target and therefore underpowered. The lower than expected incidence of severe oral mucositis in the control group further reduced power. The imbalance in the proportion of subjects receiving concurrent chemotherapy was another limitation, which likely resulted in an underestimation of the efficacy of Dentoxol. The possibility that the control rinse used may not have been completely inactive may have further reduced the observable efficacy of Dentoxol.

In conclusion, and despite the above-mentioned limitations, the results of this phase II study demonstrate that the use of Dentoxol 5 times/day is safe and resulted in significantly fewer time-points with severe oral mucositis and a delay in the onset of severe oral mucositis, compared with a control rinse. A phase III clinical trial of Dentoxol is warranted to confirm its efficacy and address the above limitations.
